# 3-Amino-4,6-dimethyl­thieno[2,3-*b*]pyridine-2-carbonitrile

**DOI:** 10.1107/S1600536809048132

**Published:** 2009-11-21

**Authors:** Xiu-Xiu Zeng, Ting-Hong Ye, Dong-Wen Lun, Luo-Ting Yu, Li Yang

**Affiliations:** a Department of Pharmaceutical and Bioengineering, School of Chemical Engineering, Sichuan University, Chengdu 610065, People’s Republic of China; b State Key Laboratory of Biotherapy and Cancer Center, West China Hospital, West China Medical School, Sichuan University, Chengdu 610041, People’s Republic of China

## Abstract

The mol­ecule of the title compound, C_10_H_9_N_3_S, is almost planar, with a dihedral angle of 1.38 (4)° between the thio­phene and pyridine rings. In the crystal packing, mol­ecules are linked into layers parallel to the *ab* plane by inter­molecular N—H⋯N hydrogen bonds and by π⋯π stacking inter­actions involving adjacent pyridine and thio­phene rings with a centroid–centroid distance of 3.537 (3) Å.

## Related literature

For the biological properties of thieno[2,3-*b*]pyridine derivatives, see: Litvinov *et al.* (2005 ▶).
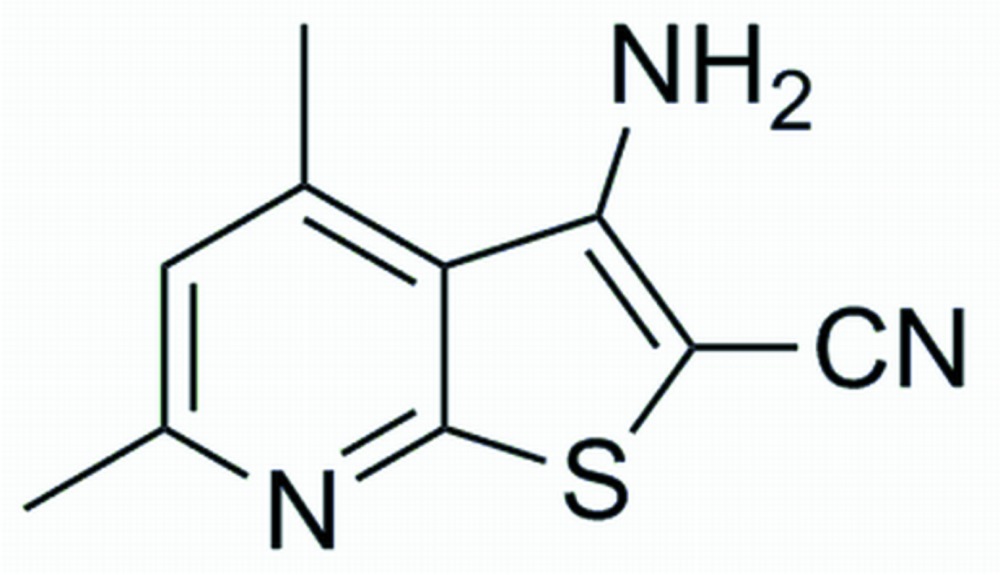


## Experimental

### 

#### Crystal data

C_10_H_9_N_3_S
*M* *_r_* = 203.26
Orthorhombic, 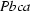

*a* = 14.562 (3) Å
*b* = 8.1252 (16) Å
*c* = 16.211 (3) Å
*V* = 1918.1 (7) Å^3^
*Z* = 8
Mo *K*α radiation
μ = 0.30 mm^−1^
*T* = 113 K
0.26 × 0.25 × 0.20 mm

#### Data collection

Rigaku Saturn CCD area detector diffractometer
Absorption correction: multi-scan (*ABSCOR*; Higashi, 1995 ▶) *T*_min_ = 0.927, *T*_max_ = 0.943
16095 measured reflections
2280 independent reflections
2047 reflections with *I* > 2σ(*I*)
*R*_int_ = 0.038

#### Refinement

*R*[*F* ^2^ > 2σ(*F* ^2^)] = 0.037
*wR*(*F* ^2^) = 0.101
*S* = 1.04
2280 reflections
137 parameters
H atoms treated by a mixture of independent and constrained refinement
Δρ_max_ = 0.33 e Å^−3^
Δρ_min_ = −0.34 e Å^−3^

### 

Data collection: *CrystalClear* (Rigaku/MSC,2005 ▶); cell refinement: *CrystalClear*; data reduction: *CrystalClear*; program(s) used to solve structure: *SHELXS97* (Sheldrick, 2008 ▶); program(s) used to refine structure: *SHELXL97* (Sheldrick, 2008 ▶); molecular graphics: *ORTEPIII* (Burnett & Johnson, 1996 ▶); software used to prepare material for publication: *PLATON* (Spek, 2009 ▶).

## Supplementary Material

Crystal structure: contains datablocks I. DOI: 10.1107/S1600536809048132/rz2391sup1.cif

Structure factors: contains datablocks I. DOI: 10.1107/S1600536809048132/rz2391Isup2.hkl

Additional supplementary materials:  crystallographic information; 3D view; checkCIF report

## Figures and Tables

**Table 1:** Hydrogen-bond geometry (Å, °)

*D*—H⋯*A*	*D*—H	H⋯*A*	*D*⋯*A*	*D*—H⋯*A*
N2—H1*N*⋯N1^i^	0.881 (19)	2.180 (19)	3.0361 (17)	163.9 (17)
N2—H2*N*⋯N3^ii^	0.89 (2)	2.35 (2)	3.0900 (18)	141.1 (16)

Symmetry codes: (i) 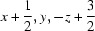; (ii) 
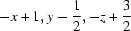
.

**Fig. 1.**
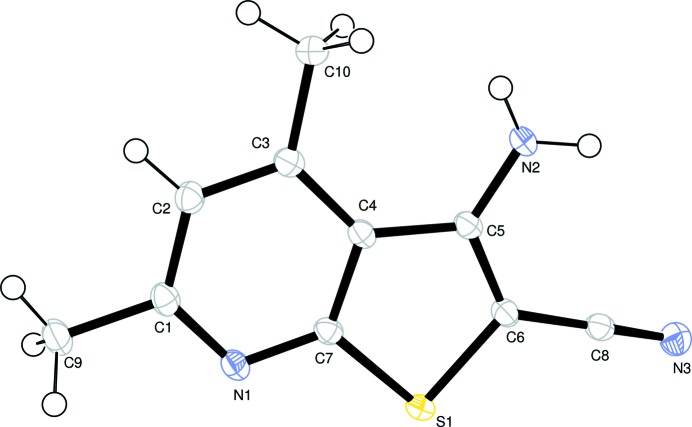
The molecular structure of the title compound, with displacement ellipsoids drawn at the 30% probability level.

**Fig. 2.**
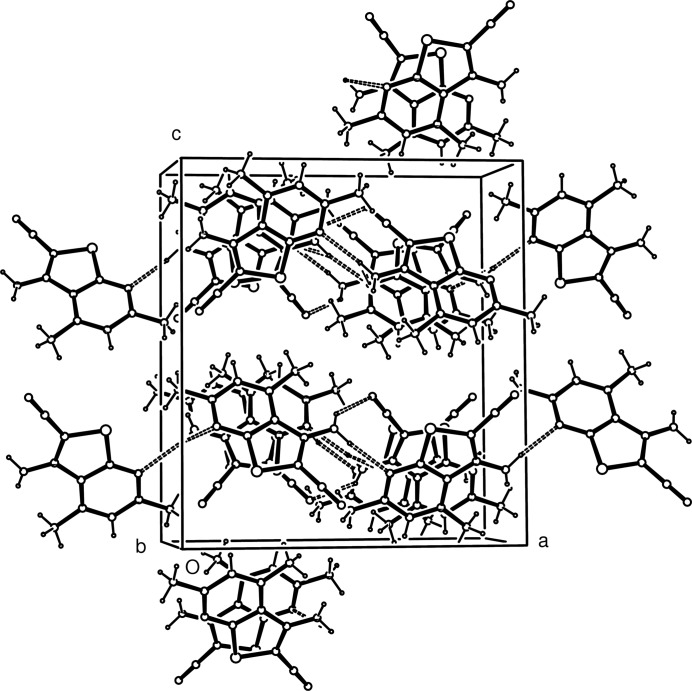
Packing diagram of the title compound. Hydrogen bonds are shown as dotted lines.

## supplementary crystallographic information

### Comment

Thieno[2,3-*b*]pyridine derivatives are of great importance owing to their wide biological properties (Litvinov *et al.*, 2005). The title compound is one of the key intermediates in our research aimed at the synthesis and investigation of new antitumor drugs. We report here its crystal structure.

The thieno[2,3-*b*]pyridine ring system of the title molecule (Fig. 1) is almost planar, the dihedral angle formed by the thiophene and pyridine rings being 1.38 (4)°. The N2 amine atom, the C8/N3 nitrile atoms and the C9/C10 methyl atoms are displaced from the mean plane through the dimethylthieno[2,3-*b*]pyridine ring system by 0.0761 (13), 0.0478 (14), 0.0922 (13), 0.0934 (15) and 0.0680 (15) Å, respectively. In the crystal structure (Fig. 2), the molecules are linked into layers parallel to the *ab* plane by intermolecular N—H···N hydrogen bonds (Table 1) and by π···π stacking interactions involving adjacent pyridine and thiophene rings, with a centroid-to-centroid distance of 3.537 (3) Å.

### Experimental

To a suspension of 4,6-dimethyl-3-cyanopyridine-2-(1*H*)-thione (1.7 g, 10 mmol) in DMF (20 ml) was added a 10% aqueous KOH solution (5.6 ml,10 mmol) followed by the addition of chloroacetonitrile (0.8 g, 10 mmol) at room temperature. After stirring for 10–15 min at r.t., a 10% aqueous KOH solution (5.6 ml,10 mmol) was added and the mixture heated to 358 K for 6 h. The reaction mixture was then allowed to cool to r.t., the precipitate was collected by filtration and washed with cold ethanol, then it was recrystallized from ethanol to give a white solid (1.6 g, 80% yield). Crystals suitable for X-ray analysis were obtained by slow evaporation of a methanol/dichloromethane (1:2 *v*/*v*) solution.

### Refinement

H atoms of the amino group were located in a difference map and refined freely. All other H atoms were positioned geometrically and refined using a riding model, with C—H = 0.93–0.97 Å and with *U*_iso_(H) = 1.2 *U*_eq_(C) or 1.5 *U*_eq_(C) for methyl H atoms.

### Crystal data

**Table:**

C_10_H_9_N_3_S	*F*(000) = 848
*M**_r_* = 203.26	*D*_x_ = 1.408 Mg m^−^^3^
Orthorhombic, *P**b**c**a*	Mo *K*α radiation, λ = 0.71073 Å
Hall symbol: -P 2ac 2ab	Cell parameters from 5470 reflections
*a* = 14.562 (3) Å	θ = 2.5–27.9°
*b* = 8.1252 (16) Å	µ = 0.30 mm^−^^1^
*c* = 16.211 (3) Å	*T* = 113 K
*V* = 1918.1 (7) Å^3^	Block, colourless
*Z* = 8	0.26 × 0.25 × 0.20 mm

### Data collection

**Table:**

Rigaku Saturn CCD area detector diffractometer	2280 independent reflections
Radiation source: rotating anode	2047 reflections with *I* > 2σ(*I*)
confocal	*R*_int_ = 0.038
Detector resolution: 7.31 pixels mm^-1^	θ_max_ = 27.9°, θ_min_ = 3.1°
ω and φ scans	*h* = −19→18
Absorption correction: multi-scan (*ABSCOR*; Higashi, 1995)	*k* = −10→8
*T*_min_ = 0.927, *T*_max_ = 0.943	*l* = −21→21
16095 measured reflections	

### Refinement

**Table:**

Refinement on *F*^2^	Primary atom site location: structure-invariant direct methods
Least-squares matrix: full	Secondary atom site location: difference Fourier map
*R*[*F*^2^ > 2σ(*F*^2^)] = 0.037	Hydrogen site location: mixed
*wR*(*F*^2^) = 0.101	H atoms treated by a mixture of independent and constrained refinement
*S* = 1.04	*w* = 1/[σ^2^(*F*_o_^2^) + (0.0627*P*)^2^ + 0.6561*P*] where *P* = (*F*_o_^2^ + 2*F*_c_^2^)/3
2280 reflections	(Δ/σ)_max_ = 0.001
137 parameters	Δρ_max_ = 0.33 e Å^−^^3^
0 restraints	Δρ_min_ = −0.34 e Å^−^^3^

### Special details

**Table:**

Geometry. All e.s.d.'s (except the e.s.d. in the dihedral angle between two l.s. planes) are estimated using the full covariance matrix. The cell e.s.d.'s are taken into account individually in the estimation of e.s.d.'s in distances, angles and torsion angles; correlations between e.s.d.'s in cell parameters are only used when they are defined by crystal symmetry. An approximate (isotropic) treatment of cell e.s.d.'s is used for estimating e.s.d.'s involving l.s. planes.
Refinement. Refinement of *F*^2^ against ALL reflections. The weighted *R*-factor *wR* and goodness of fit *S* are based on *F*^2^, conventional *R*-factors *R* are based on *F*, with *F* set to zero for negative *F*^2^. The threshold expression of *F*^2^ > σ(*F*^2^) is used only for calculating *R*-factors(gt) *etc*. and is not relevant to the choice of reflections for refinement. *R*-factors based on *F*^2^ are statistically about twice as large as those based on *F*, and *R*- factors based on ALL data will be even larger.

### Fractional atomic coordinates and isotropic or equivalent isotropic displacement parameters (Å^2^)

**Table:**

	*x*	*y*	*z*	*U*_iso_*/*U*_eq_	
S1	0.20101 (2)	0.66501 (4)	0.69746 (2)	0.01744 (13)	
N1	0.09825 (8)	0.51450 (14)	0.81134 (7)	0.0168 (3)	
N2	0.43428 (8)	0.53762 (16)	0.80376 (7)	0.0197 (3)	
H1N	0.4779 (13)	0.550 (2)	0.7666 (11)	0.030 (5)*	
H2N	0.4479 (13)	0.463 (2)	0.8416 (12)	0.032 (5)*	
N3	0.42910 (9)	0.79977 (16)	0.61222 (7)	0.0231 (3)	
C1	0.09139 (9)	0.42573 (17)	0.88087 (8)	0.0175 (3)	
C2	0.16908 (9)	0.37944 (17)	0.92658 (8)	0.0179 (3)	
H2	0.1608	0.3189	0.9762	0.021*	
C3	0.25762 (9)	0.41919 (17)	0.90166 (8)	0.0163 (3)	
C4	0.26566 (9)	0.50818 (16)	0.82730 (8)	0.0150 (3)	
C5	0.34563 (9)	0.56490 (16)	0.78168 (8)	0.0154 (3)	
C6	0.31997 (10)	0.65374 (17)	0.71273 (9)	0.0170 (3)	
C7	0.18349 (9)	0.55260 (17)	0.78720 (8)	0.0151 (3)	
C8	0.38019 (9)	0.73372 (17)	0.65747 (8)	0.0175 (3)	
C9	−0.00311 (9)	0.37597 (18)	0.90758 (9)	0.0222 (3)	
H9A	−0.0352	0.4716	0.9305	0.033*	
H9B	0.0013	0.2899	0.9497	0.033*	
H9C	−0.0373	0.3338	0.8600	0.033*	
C10	0.33843 (10)	0.36632 (19)	0.95308 (8)	0.0225 (3)	
H10A	0.3165	0.3099	1.0027	0.034*	
H10B	0.3743	0.4633	0.9690	0.034*	
H10C	0.3772	0.2913	0.9210	0.034*	

### Atomic displacement parameters (Å^2^)

**Table:**

	*U*^11^	*U*^22^	*U*^33^	*U*^12^	*U*^13^	*U*^23^
S1	0.0131 (2)	0.0222 (2)	0.0170 (2)	0.00032 (12)	−0.00226 (11)	0.00211 (12)
N1	0.0128 (6)	0.0185 (6)	0.0190 (5)	0.0002 (4)	0.0006 (4)	−0.0026 (4)
N2	0.0116 (6)	0.0276 (7)	0.0198 (6)	0.0016 (5)	0.0007 (4)	0.0031 (5)
N3	0.0209 (6)	0.0269 (7)	0.0217 (6)	−0.0034 (5)	0.0009 (5)	0.0004 (5)
C1	0.0153 (7)	0.0178 (6)	0.0194 (6)	−0.0003 (5)	0.0022 (5)	−0.0038 (5)
C2	0.0175 (7)	0.0198 (7)	0.0165 (6)	0.0004 (5)	0.0025 (5)	−0.0011 (5)
C3	0.0154 (6)	0.0175 (6)	0.0159 (6)	0.0016 (5)	−0.0006 (5)	−0.0035 (5)
C4	0.0127 (6)	0.0158 (6)	0.0165 (6)	0.0001 (5)	0.0000 (5)	−0.0032 (5)
C5	0.0129 (6)	0.0166 (6)	0.0166 (6)	0.0003 (5)	−0.0008 (5)	−0.0031 (5)
C6	0.0131 (6)	0.0201 (7)	0.0178 (6)	−0.0005 (5)	−0.0003 (5)	−0.0011 (5)
C7	0.0140 (6)	0.0155 (6)	0.0157 (6)	0.0004 (5)	−0.0012 (5)	−0.0023 (5)
C8	0.0156 (6)	0.0194 (6)	0.0175 (6)	0.0004 (5)	−0.0026 (5)	−0.0019 (5)
C9	0.0141 (7)	0.0269 (8)	0.0255 (7)	−0.0019 (5)	0.0028 (5)	−0.0012 (6)
C10	0.0180 (7)	0.0316 (8)	0.0179 (6)	0.0027 (6)	−0.0012 (5)	0.0037 (6)

### Geometric parameters (Å, °)

**Table:**

S1—C7	1.7366 (14)	C3—C4	1.4104 (18)
S1—C6	1.7522 (15)	C3—C10	1.5047 (19)
N1—C7	1.3377 (17)	C4—C7	1.4088 (18)
N1—C1	1.3419 (17)	C4—C5	1.4546 (18)
N2—C5	1.3579 (17)	C5—C6	1.3820 (19)
N2—H1N	0.881 (19)	C6—C8	1.4121 (19)
N2—H2N	0.89 (2)	C9—H9A	0.9800
N3—C8	1.1547 (18)	C9—H9B	0.9800
C1—C2	1.4038 (19)	C9—H9C	0.9800
C1—C9	1.4982 (18)	C10—H10A	0.9800
C2—C3	1.3892 (19)	C10—H10B	0.9800
C2—H2	0.9500	C10—H10C	0.9800
			
C7—S1—C6	89.97 (7)	C5—C6—C8	125.84 (13)
C7—N1—C1	116.05 (11)	C5—C6—S1	114.14 (10)
C5—N2—H1N	119.1 (12)	C8—C6—S1	120.02 (11)
C5—N2—H2N	120.5 (12)	N1—C7—C4	126.42 (12)
H1N—N2—H2N	112.9 (16)	N1—C7—S1	120.21 (10)
N1—C1—C2	121.83 (12)	C4—C7—S1	113.36 (10)
N1—C1—C9	117.14 (12)	N3—C8—C6	179.64 (15)
C2—C1—C9	121.03 (12)	C1—C9—H9A	109.5
C3—C2—C1	122.15 (13)	C1—C9—H9B	109.5
C3—C2—H2	118.9	H9A—C9—H9B	109.5
C1—C2—H2	118.9	C1—C9—H9C	109.5
C2—C3—C4	116.41 (12)	H9A—C9—H9C	109.5
C2—C3—C10	119.88 (12)	H9B—C9—H9C	109.5
C4—C3—C10	123.71 (12)	C3—C10—H10A	109.5
C7—C4—C3	117.08 (12)	C3—C10—H10B	109.5
C7—C4—C5	111.36 (12)	H10A—C10—H10B	109.5
C3—C4—C5	131.56 (12)	C3—C10—H10C	109.5
N2—C5—C6	123.75 (13)	H10A—C10—H10C	109.5
N2—C5—C4	125.13 (12)	H10B—C10—H10C	109.5
C6—C5—C4	111.11 (12)		
			
C7—N1—C1—C2	1.59 (19)	N2—C5—C6—C8	−2.6 (2)
C7—N1—C1—C9	−177.76 (12)	C4—C5—C6—C8	176.41 (13)
N1—C1—C2—C3	−1.6 (2)	N2—C5—C6—S1	178.35 (11)
C9—C1—C2—C3	177.76 (13)	C4—C5—C6—S1	−2.65 (15)
C1—C2—C3—C4	−0.4 (2)	C7—S1—C6—C5	1.57 (11)
C1—C2—C3—C10	−179.98 (13)	C7—S1—C6—C8	−177.55 (12)
C2—C3—C4—C7	2.05 (18)	C1—N1—C7—C4	0.3 (2)
C10—C3—C4—C7	−178.37 (12)	C1—N1—C7—S1	179.24 (10)
C2—C3—C4—C5	−177.59 (13)	C3—C4—C7—N1	−2.2 (2)
C10—C3—C4—C5	2.0 (2)	C5—C4—C7—N1	177.56 (13)
C7—C4—C5—N2	−178.43 (13)	C3—C4—C7—S1	178.83 (10)
C3—C4—C5—N2	1.2 (2)	C5—C4—C7—S1	−1.46 (15)
C7—C4—C5—C6	2.60 (16)	C6—S1—C7—N1	−179.09 (12)
C3—C4—C5—C6	−177.75 (14)	C6—S1—C7—C4	0.00 (11)

### Hydrogen-bond geometry (Å, °)

**Table:**

*D*—H···*A*	*D*—H	H···*A*	*D*···*A*	*D*—H···*A*
N2—H1N···N1^i^	0.881 (19)	2.180 (19)	3.0361 (17)	163.9 (17)
N2—H2N···N3^ii^	0.89 (2)	2.35 (2)	3.0900 (18)	141.1 (16)

Symmetry codes: (i) *x*+1/2, *y*, −*z*+3/2; (ii) −*x*+1, *y*−1/2, −*z*+3/2.
